# Deep Cooperative Spectrum Sensing Based on Residual Neural Network Using Feature Extraction and Random Forest Classifier

**DOI:** 10.3390/s21217146

**Published:** 2021-10-28

**Authors:** Myke D. M. Valadão, Diego Amoedo, André Costa, Celso Carvalho, Waldir Sabino

**Affiliations:** 1 Center for R&D in Electronic and Information Technology (CETELI), Department of Electronics and Computing (DTEC), Federal University of Amazonas (UFAM), Manaus 69067005, Brazil; ccarvalho_@ufam.edu.br (C.C.); waldirjr@ufam.edu.br (W.S.); 2 National Telecommunications Agency (ANATEL), Manaus 69057070, Brazil; diegoalves@anatel.gov.br; 3 Electronic and Telecommunications Engineering, Faculty of Electrical Engineering (FEELT), Federal University of Uberlândia (UFU), Uberlandia 38408288, Brazil; alacosta@ufu.br

**Keywords:** cooperative spectrum sensing, residual neural network, cognitive radio

## Abstract

Some bands in the frequency spectrum have become overloaded and others underutilized due to the considerable increase in demand and user allocation policy. Cognitive radio applies detection techniques to dynamically allocate unlicensed users. Cooperative spectrum sensing is currently showing promising results. Therefore, in this work, we propose a cooperative spectrum detection system based on a residual neural network architecture combined with feature extractor and random forest classifier. The objective of this paper is to propose a cooperative spectrum sensing approach that can achieve high accuracy in higher levels of noise power density with less unlicensed users cooperating in the system. Therefore, we propose to extract features of the sensing information of each unlicensed user, then we use a random forest to classify if there is a presence of a licensed user in each band analyzed by the unlicensed user. Then, information from several unlicensed users are shared to a fusion center, where the decision about the presence or absence of a licensed user is accomplished by a model trained by a residual neural network. In our work, we achieved a high level of accuracy even when the noise power density is high, which means that our proposed approach is able to recognize the presence of a licensed user in 98% of the cases when the evaluated channel suffers a high level of noise power density (−134 dBm/Hz). This result was achieved with the cooperation of 10 unlicensed users.

## 1. Introduction

Recently, with increased demand and the next generation of communication systems, such as 5G and 6G, the policy of allocation of users in the frequency spectrum, proposed by the International Telecommunications Union (ITU), has become inefficient [1,2,3]. Because of this policy, some spectrum bands are overloaded and others underutilized as cited by [4,5]. The cognitive radio (CR) applies spectrum sensing (SS) techniques to dynamically allocate unlicensed users (UU) in spectrum holes in order to increase efficiency of the spectrum [6,7]. There are two main SS approaches, the narrowband and wideband sensing approaches [8]. In the narrowband approach, each frequency channel is analyzed at a time, differently from the wideband approach, where many channels are analyzed sequentially or simultaneously [9], although the sequential method requires a longer time and more consumption of energy, which is not ideal for real-time communication systems [10].

Among the most popular narrowband techniques, we have: energy detection, where usually the presence of licensed user (LU) is determined when the value of the power of the received signal is above a threshold, or in the absence of the LU when the power of the received signal is below the threshold [11,12]; cyclostationary detection, when the periodic features of the received signal can be used for its detection [12,13]; matched filter detection, this detection is made by comparing the received signal with a saved pilot samples, then a threshold is apply to determinate the presence or not of LU [14,15]; and covariance-based detection, where the covariance matrix of the received signal is used to determinate the presence of LU [16].

The UU, due to the high data rates required by the next generation of communication systems, needs to sense a considerable range of spectrum frequencies, several wideband SS techniques have been proposed [17]. Among these techniques, sensing techniques based on Nyquist and sub-Nyquist have shown promise, as some authors point out in [18]. In Nyquist wideband sensing techniques, the received signal at some point is sampled by a traditional analog-to-digital converter with a Nyquist sampling rate ($f_{s}>2f_{m}$) [19]; as an advantage, these techniques present a simple structure, and as disadvantages, high sampling rate and energy cost [20]. The sub-Nyquist-based techniques overcome the disadvantages presented in Nyquist-based SS by reducing the sampling rate and then detecting spectral holes with the partial data that remained [21]; as an advantage, these techniques present a low sampling rate, and as disadvantage, they are sensitive to design imperfections [22]. More recently some wideband SS approaches shown to be promising, such as cooperative spectral sensing (CSS) and some approaches using deep learning networks [23].

The CSS consists in combining the wideband sensing information of multiple UU in order to increase the probability of correct identification of the LU [24,25,26]. Along with CSS, approaches involving deep learning networks have shown promise to identify LU in the spectrum, and recurrent neural networks (RNN) and convolutional neural networks (CNN) are recently the most proposed classification methods for SS [27]. For instance, in [28], they proposed a CSS approach using an energy detection technique and a supervised CNN to determine the presence, or not, of LU. In [24], they proposed a CNN to identify the presence of LU based in a spatial–temporal dataset. Furthermore, in [29], they proposed a Long Short-Term Memory (LSTM) based deep learning model to predict the multi-channel conditions.

In this article, we present an investigation into CSS based on machine learning and deep learning. We propose a random forest (RF) classifier and a residual convolutional neural network (ResNet) to detect the presence of LU. Unlike some authors, our proposal focuses on reducing complexibility by extracting features of received signals, similar to that proposed by [30]. In the performance evaluation process, we evaluate the accuracy of the models generated in relation to noise power density ($N_{0}$) and the number of UU, also a response time evaluation of the entire sensing process is made. The proposed method takes into consideration higher levels of $N_{0}$ and yet achieves considerable good results, as when the $N_{0}$ is lower than −134 dBm/Hz, the accuracy reaches more than 99% and the computation time is below 0.05 seconds even with great number of UU.

In this work, a system is presented where in adverse channel conditions it is possible to identify a LU, and thus better allocate UU in spectral holes. The results presented good accuracy even in high levels of $N_{0}$, which gives the proposed system better reliability even in adverse conditions. To achieve the results presented, we extracted 10 features from the signals received by each UU to highlight important signal characteristics and used a random forest classifier, that segregated the two classes based on entropy. Then, with detection information from many cooperating UUs, we train a ResNet to generate a model that can decide whether the channels evaluated by the UU have LU presence or not. The main contributions of this article can be summarized as follows:

- When generating the signals, severe noise conditions were taken into account, such as the wide range of distances between users and the high noise level itself. In addition, a large amount of instances was generated.
- The spectral and transformation features were extracted to represent the signal received by each UU in a vector, reducing needs for a complex classification model.
- The proposed ResNet and some deep learning approaches such as CNN and RNN were trained and tested, as well as classical machine learning algorithms such as RF and support vector machine (SVM). The corresponding accuracy of these networks is analyzed and compared.
- A high level of accuracy in the correct identification of LUs was achieved by taking into account the high level of $N_{0}$ and the cooperation of few UUs. Therefore, the experimental results validate the effectiveness of the proposed scheme.

This paper is organized as follows. In Section 2 we present the related works. Then, material and methods are presented in Section 3, followed by the experiments and results in Section 4. In Section 5, we present the discussion about the results found. Finally, in Section 6, the main conclusions are derived from this study and we suggest possible future work.

## 2. Related Works

In [28], the authors proposed an energy detector along with a CNN for CSS. In their work, a single LU and several UU move randomly across an area with some speed over a period of time. Then, a hard decision approach (HD) and a soft decision approach (SD) for an energy detector is used to identify if there is an LU presence in the evaluated channel. Then, the information of many UU in the system is shared to a fusion center where a pre-trained model is used to identify if in the evaluated frequency ranges there is the presence of LU. This pre-trained model was generated by a CNN with convolutional and fully connected parts. The convolution part is composed of a sequence of three sub-blocks each containing a 3×3 convolution layer, activation function, and max pooling 2D. They obtained good results with the increase in the number of unlicensed users, although the $N_{0}$ in their experiments is low. In comparison with the proposed method in [28], we presented a method were we achieve similar accuracy rate and time processing with less UU in the system and with a higher level of $N_{0}$, showing more reliability from our method.

In [31], the authors also proposed an energy detector for detecting spectral holes, although in this work the authors used an RF classifier to train a model to identify the presence of LU. Its energy detection model is based on a threshold, if the signal strength is above this threshold then the algorithm considered that in the evaluated channel there is the presence of LU. In phase two, they did a data augmentation to down-sampling and over-sampling the data, specifically, they used the synthetic minority over-sampling (SMOTE) technique. Then a supervised RF classifier is used to train a model. As a result they obtained 91% accuracy with SMOTE set to 50:50, although the authors provide little information about the conditions for generating the signals. In the first stage of the our proposed method, in comparison with the presented by [31], we present a accuracy rate superior to 91% using also a RF classifier to identify the presence of LU in the channels.

In [22], the authors proposed a DLSenseNet (spectrum detection network based on deep learning) as a deep learning approach to identify channel conditions. They used the RadioML2016.10b dataset for the study. Its neural network is composed of three blocks, the inc block, the LSTM block, and the dense block. The inc block is made up of three parallel paths with different filter sizes, the LSTM block contains 128 cells and then the dense part is made up of some fully connected layers. We can note in their work that they achieve good results even in a lower signal-to-noise ratio (SNR), and compared to other methods, they have higher detection probability with the proposed DLSenseNet. Comparing with our paper, the method proposed by [22] also used a deep neural network to evaluated the conditions of the channel, but do not provide information of response time evaluation, and do not show if the proposed method is applicable to real scenarios.

In [6], the authors made an analysis of the spectrum sensing techniques such as energy detection, matched filtering detection, eigenvalue-based detection and cyclostationary detection. A discussion about CSS was also made. They focused on practical sensing algorithm designs, and blind sensing methods that do not need the information of the source signal and conditions of the propagation channel. They reached the conclusion that space-time joint signal processing increases the sensing performance and solves the noise uncertainty problem in some cases. Related to CSS, the authors pointed to some challenges about the method of decision in the fusion center. They pointed out that in most literature, the simple energy detection technique is used, which may not be robust enough. In comparison, our proposed method is based in a trained model with several examples of signals in multiple channel conditions.

The authors in [25] proposed a CSS approach to overcome the problem of the conditions of the received signals. In their work, they described the interference range and detection sensitivity. They used an energy detection function to determinate if the channel is occupied or not by the LU in a local process. A cooperative sensing with a trade-off regularization is proposed to avoid consumption of time and increase probability of detection. Their results showed that, already with the cooperation of four UU, the probability of false alarm decreased, with more UU cooperating in the system the probability of detection is increased because, intuitively, some users will have a channel that is significantly better. In our approach, we used methods based on machine learning and deep learning to reduce the probability of error and, with a few UUs cooperating, we achieve a high level of accuracy.

The authors in [26] proposed a CSS scheme to optimize the sensing performance. They used an energy detection technique and proposed a half-voting rule as an optimal fusion rule. They analyzed the performance of the method with the variation of the threshold of the energy detector in order to find the best value. Furthermore, they also analyzed the probability of error with the threshold values and the variation of UUs in the system. It was concluded that the half-voting rule is efficient to increase performance, and depending on the threshold, the AND rule or the OR rule is more efficient. Different from this method, our method of decision is made by a deep learning trained model with several signals generated in multiple conditions.

Lastly, in [24], they proposed a CSS approach generating a dataset with 25 UU and using a CNN in two scenarios. The data were generated taking into consideration the in-phase and quadrature components and frame size, so the data have the shape 25×128×2 for all the scenarios. In the first scenario, the proposed CNN was composed of two convolution layers followed by *flatten* and *dense* layers. For the second scenario, a more robust CNN was proposed, and this CNN was built with a two convolution layers with *dropout* in each one, then a *flatten* layer followed by two *dense* layers. As result, in [21], they achieved good results for higher levels of $N_{0}$ with the cooperation of 25 UU. We propose a system that achieves a similar accuracy rate with fewer UU.

## 3. Proposed Deep Cooperative Spectrum Sensing Using Random Forest and Residual Neural Network

### 3.1. Setup

The experiments were done on an Acer Intel(R) Core (TM) i7-7700HQ CPU @ 2.81 GHz notebook with 16 GB of RAM and NVIDIA GeForce GTX 1050 Ti in Windows 10 environment. The algorithms of the experiments were implemented in Python version 3.7.9.

### 3.2. System Model

We propose a CSS approach where each individual UU shares the detection information with a fusion center where a model will decide whether the channels evaluated by these UUs have LU presence or not. For this proposal, we divided the methodological process into two phases. The first phase is the generation of the dataset, and for this task, we generate the signals, extract features and apply a RF classifier to generate a model that can identify whether in the individual channel evaluated there is the presence of LU. Then, in phase two, we collect all the information of many UUs cooperating to generate matrices with variation of UUs, the number of bands evaluated by each UU is fixed, and these matrices are the input data of an ResNet network with the goal to generate a model that can identify, in wideband, more precisely the presence of LU with the cooperation of UUs.

In the generation of signals, we assume two hypotheses, the signal noise and the LU signal. Thus, we generate signals taking into consideration a few variables. We assumed that the UUs and the LU move at the same speed and their start location are randomly chosen in a certain area, then the location of the users changes over time. The bandwith, the noise power density and the multipath fading are also variables taken into account. Thus, from the generated signals, we extract spectral and transform features to highlight the singular characteristics of the signals to facilitate the classification process and reduce computational cost. Then, each vector of features represents a signal, that can be noise or LU signal, that will be used to train a RF classifier in order to generate a model for individual SS. Therefore, each UU will extract features of the received signals and apply the RF model to determinate if there is the presence, or not, of LU in each individual channel evaluated. The output of the first phase of the proposed method will be a vector containing the information of the condition of the evaluate channels for each UU.

**Figure 1:**
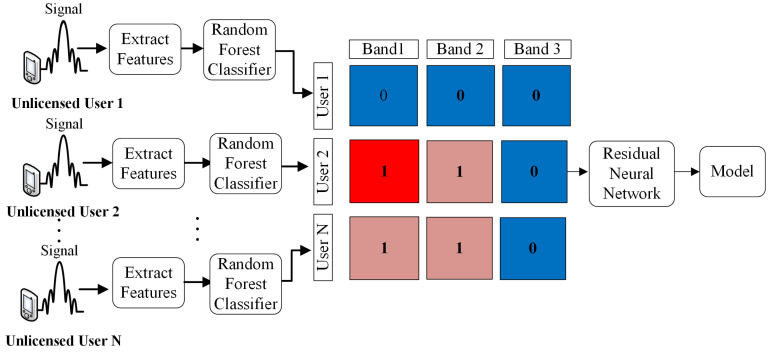
Proposed system for generation of the CSS model.

The second phase is related with the CSS process. Starting with the training of a ResNet network using the output of the first phase. So, several matrices with the information sensing of many UUs are used as inputs to train the propose network. The model generated is used in the fusion center to determinate if the channels sensed by the UUs under different conditions the presence of LU. Therefore, the hypothesis is that with the cooperation of several UUs in the system, the probability of identify spectral holes will increase. In Figure 1, we show a block diagram of the proposed method.

**Notation** **1.** *In this section, we present the notations used in this work, as well as some examples of their uses. Discrete signals (or vectors) are defined by variables without bold and lowercase followed by parentheses, such as y(n), s(n), f(n), and a(n). The variable ϕ is specific to phase. In some cases, we use indexes i, j, n, and nc as in $y_{i}^{j}\left(n\right)$ to represent unlicensed users i on band j. The $H_{0}$ and $H_{1}$ are specific variables that define the decisions that the system, designed for SS, can make. Variables in cal followed by braces are used for transforms, as in DFT{.} and DWT{.}. In Section 3.3.2, we define all the features used in this work. Each feature follows the standard notation used in the literature. A special case of notation occurs in some equations in this section. Note that, considering a vector v(n), the term*

$$\sum_{a_{n}\left(n\right)\geq 0.1}v\left(n\right)$$

*is an specific notation to indicate that the summation of v(n) samples is performed only on the values of $a_{n}\left(n\right)\geq 0.1$. In other words, $a_{n}\left(n\right)\geq 0.1$ is a threshold that separate non-weak and weak samples of the vector v(n).*

### 3.3. Dataset

#### 3.3.1. Signal Generation

In the SS process, the decision on the condition of the channel is binary, and two hypotheses should be considered, $H_{1}$ and $H_{0}$, where $H_{1}$ represents the hypothesis in which the LU is present and $H_{0}$ when the LU is not present. For building the signals that represent these two hypotheses, we assume that $N_{UU}$ UUs and a single LU move at a speed *v* and their starting positions are randomly chosen in a given area, so that the users’ location changes over a period of time Δt. We also considered a multi-channel system with $N_{B}$ bands whose bandwidth is $B_{W}$. Furthermore, we assume that the UU is not aware of which bands are used by the LU and the LU can use $N_{B_{P}}$ consecutive bands. So, the received signal of UU *i* on band *j* at time *n* can be described as

(1) $$y_{i}^{j}\left(n\right)=\left\{\begin{array}{cc} s_{i}^{j}\left(n\right)+w_{i}^{j}\left(n\right), & \mathrm{for}H_{1}\mathrm{and}j\in B_{P}\\ \sqrt{\eta }s_{i}^{j}\left(n\right)+w_{i}^{j}\left(n\right), & \mathrm{for}H_{1}\mathrm{and}j\in B_{A}\\ w_{i}^{j}\left(n\right), & \mathrm{for}H_{0} \end{array}\right.$$

where $s_{i}^{j}\left(n\right)=\kappa_{i}\left(n\right)g_{i}^{j}\left(n\right)x\left(n\right)$ and $w_{i}^{j}\left(n\right)$ is the additive white Gaussian noise (AWGN) whose noise power density is $N_{0}$, mean zero and standard deviation $\sigma =\sqrt{B_{W}10^{\frac{N_{0}}{10}}}$. Being η the proportion of power leaked to adjacent bands, then $B_{P}$ are the bands occupied by the LU and $B_{A}$ are the bands affected by the leaked power of the LU.

In the $s_{i}^{j}\left(n\right)$, we have the simplified path loss model that can be written as

(2) $$\kappa_{i}\left(n\right)=\sqrt{\frac{P}{\beta {\left(d_{i}\left(n\right)\right)}^{\alpha }10^{\frac{h_{i}\left(n\right)}{10}}}}$$

where α and β are the path-loss exponent and path-loss constant, respectively. Let $d_{i}\left(n\right)$ be the Euclidean distance between the LU and UU *i* at time *n*. The shadow fading of the channel, $h_{i}\left(n\right)$, between the the LU and the UU *i* at time *n* in dB can be described as a normal distribution with mean zero and variance $\sigma^{2}$, and *P* is the power transmitted by the LU in a given band. Furthermore, the multipath fading, $g_{i}^{j}\left(n\right)$, is modeled as a independent zero-mean circularly symmetric complex Gaussian (CSCG) random variable. Furthermore, x(n) is the data transmitted by the LU at time *n* with expected value of x(n) equal to one.

#### 3.3.2. Feature Extraction

In order to reduce the complexity of the system, we propose extracting features from the received signals that highlight important proprieties to discriminate signals [32]. The features and their descriptions are below:

- Maximum value of the power spectrum density (PSD) of the normalized and centralized instantaneous amplitude ($\gamma_{max}$):
  (3) $$\gamma_{max}=\frac{\max|\mathrm{DFT}\left\{a_{nc}\left(n\right)\right\}{|}^{2}}{N_{s}}$$
  where $N_{s}$ the number of samples by segment and $a_{nc}\left(n\right)$ is the normalized and centralized instantaneous amplitude, $a_{nc}\left(n\right)=\frac{|H\left\{y(n)\right\}e^{i2\pi f_{c}n}|}{m_{a}}-1$. Being $H\left\{y(n)\right\}$ the Hilbert transform, y(n) the received signal sampled at $t=\frac{n}{fs}$ and $m_{a}$ is given by $\frac{1}{N_{s}}\sum_{n=1}^{N_{s}}\left|H\left\{y(n)\right\}e^{i2\pi f_{c}n}\right|$.
- Standard deviation of the normalized and centralized instantaneous amplitude ($\sigma_{aa}$):
  (4) $$\sigma_{aa}=\sqrt{\frac{1}{N_{s}}\sum_{n=1}^{N_{s}}{\left(a_{nc}\left(n\right)-\bar{a_{nc}\left(n\right)}\right)}^{2}}$$
  where $\bar{a_{nc}\left(n\right)}$ is the average of the normalized and centralized instantaneous amplitude.
- Standard deviation of the centralized nonlinear absolute instantaneous phase ($\sigma_{ap}$) is evaluated over non-weak ranges of the signal segment. The weak segments refer to values of the amplitude, $a_{n}$, that are susceptible to phase distortions due to the insertion of Gaussian noise, then the region where $a_{n}\left(n\right)\geq 0.1$ as non-weak segments was defined. The $\sigma_{ap}$ is expressed below:
  (5) $$\sigma_{ap}=\sqrt{\frac{1}{C}\left(\sum_{a_{n}\left(n\right)\geq 0.1}\phi_{NL}^{2}\left(n\right)\right)-{\left(\sum_{a_{n}\left(n\right)\geq 0.1}\left|\phi_{NL}\left(n\right)\right|\right)}^{2}}$$
  where $a_{n}\left(n\right)=\frac{|H\left\{y(n)\right\}e^{i2\pi f_{c}n}|}{m_{a}}$ and *C* is the total of samples in the non-weak segment of the signal. The variable $\phi_{NL}$ is the nonlinear phase described by angulation between the real and imaginary components of the Hilbert transform of the received signal $H\left\{y(n)\right\}$. Furthermore, $\phi_{NL}\left(n\right)$ is the value of the nonlinear component of the instantaneous phase in instants of time $t=\frac{n}{f_{s}}$.
- Standard deviation of the centralized direct nonlinear phase ($\sigma_{dp}$):
  (6) $$\sigma_{dp}=\sqrt{\frac{1}{C}\left(\sum_{a_{n}\left(n\right)\geq 0.1}\phi_{NL}^{2}\left(n\right)\right)-{\left(\sum_{a_{n}\left(n\right)\geq 0.1}\phi_{NL}\left(n\right)\right)}^{2}}$$
- Standard deviation of normalized and centralized instantaneous frequency ($\sigma_{af}$) is evaluated over non-weak ranges of a signal segment, $\sigma_{af}$ is obtained according to the following expression:
  (7) $$\sigma_{af}=\sqrt{\frac{1}{C}\left(\sum_{a_{n}\left(n\right)\geq 0.1}f_{N}^{2}\left(n\right)\right)-{\left(\sum_{a_{n}\left(n\right)\geq 0.1}f_{N}\left(n\right)\right)}^{2}}$$
  being $f_{N}\left(n\right)=\frac{f\left(n\right)-m_{f}}{r_{s}}$, where $r_{s}$ is the digital sequence symbol rate, $m_{f}=\frac{1}{N_{s}}\sum_{n=1}^{N_{s}}f\left(n\right)$ and f(n) is the instantaneous frequency given by the derivative relative to the time of $\phi_{NL}\left(n\right)$ divided by 2π, $\frac{1}{2\pi }\frac{d\phi_{NT}}{dt}$.
- Standard deviation of the absolute value of the normalized and centralized instantaneous frequency ($\sigma_{f}$):
  (8) $$\sigma_{f}=\sqrt{\frac{1}{C}\left(\sum_{a_{n}\left(n\right)\geq 0.1}f_{N}^{2}\left(n\right)\right)-{\left(\sum_{a_{n}\left(n\right)\geq 0.1}\left|f_{N}\left(n\right)\right|\right)}^{2}}$$
- Maximum PSD value of normalized and centralized instantaneous frequency ($\gamma_{maxf}$) is given by the equation:
  (9) $$\gamma_{maxf}=\frac{\max|\mathrm{DFT}\left\{f_{N}\left(n\right)\right\}{|}^{2}}{N_{s}}$$
- Maximum value of the discrete cosine transform ($max_{dct}$):
  (10) $$C_{x}\left(k\right)=\left\{\begin{array}{cc} \sum_{n=0}^{N-1}2H\left\{y(n)\right\}\cos\left(\frac{\pi }{2N}k\left(2n+1\right)\right), & for0\leq k\geq N\\ 0, & \mathrm{otherwise} \end{array}\right.$$
  The maximum value resulting from the use of the discrete cosine transform over the complex wrap of the signal, given by the $H\left\{y(n)\right\}$, represents the feature.
- Maximum value of the Walsh–Hadamard Transform ($\sigma_{wht}$):
  (11) $${\mathrm{WTH}}_{N}=\begin{array}{c} n\mathrm{times}\\ \overbrace{{\mathrm{DFT}}_{2}⨂\cdots ⨂{\mathrm{DFT}}_{2}} \end{array}$$
  being ${\mathrm{DFT}}_{2}=\left[\begin{array}{cc} 1 & 1\\ 1 & -1 \end{array}\right]$ the DFT matrix of two points and ⨂ the Kronecker product. The feature is obtained by calculating the maximum value of the coefficients of the Walsh–Hadamard transform of the complex wrap of the signal.
- Standard deviation of the discrete Wavelet transform ($\sigma_{dwt}$):
  (12) $$\sigma_{dwt}=\sqrt{\frac{1}{N_{s}}\sum_{n=1}^{N_{s}}{\left(\mathrm{DWT}\left\{H\left\{y(n)\right\}\right\}-\sum_{n=1}^{N_{s}}\frac{\mathrm{DWT}\{H\left\{y(n)\right\}\}}{n}\right)}^{2}}$$
  where DWT is the discrete Wavelet transform.

#### 3.3.3. Random Forest Classifier

The RF is a supervised learning algorithm initially proposed in 2001, and has been widely used in classification and regression tasks [33]. The *forest* is built with several decision trees, usually trained with the *bagging* method, and merges them to reduce variance compared to a single decision tree, overcoming problems such as overfitting. Bagging increases the accuracy and stability of prediction by combining learning models [33]. For the proposed methodology, we assume the default of *scikit-learn* library, of which the boosting method is the default. Weighted voting is used in the boosting method [31]. While bagging method does not depend on previous model results, boosting method depends on the performance on the previous models. The structure of each individual tree, the structure and size of the *forest*, and the randomness of the RF are controlled by hyperparameters.

### 3.4. Residual Convolutional Neural Network

**Figure 2:**
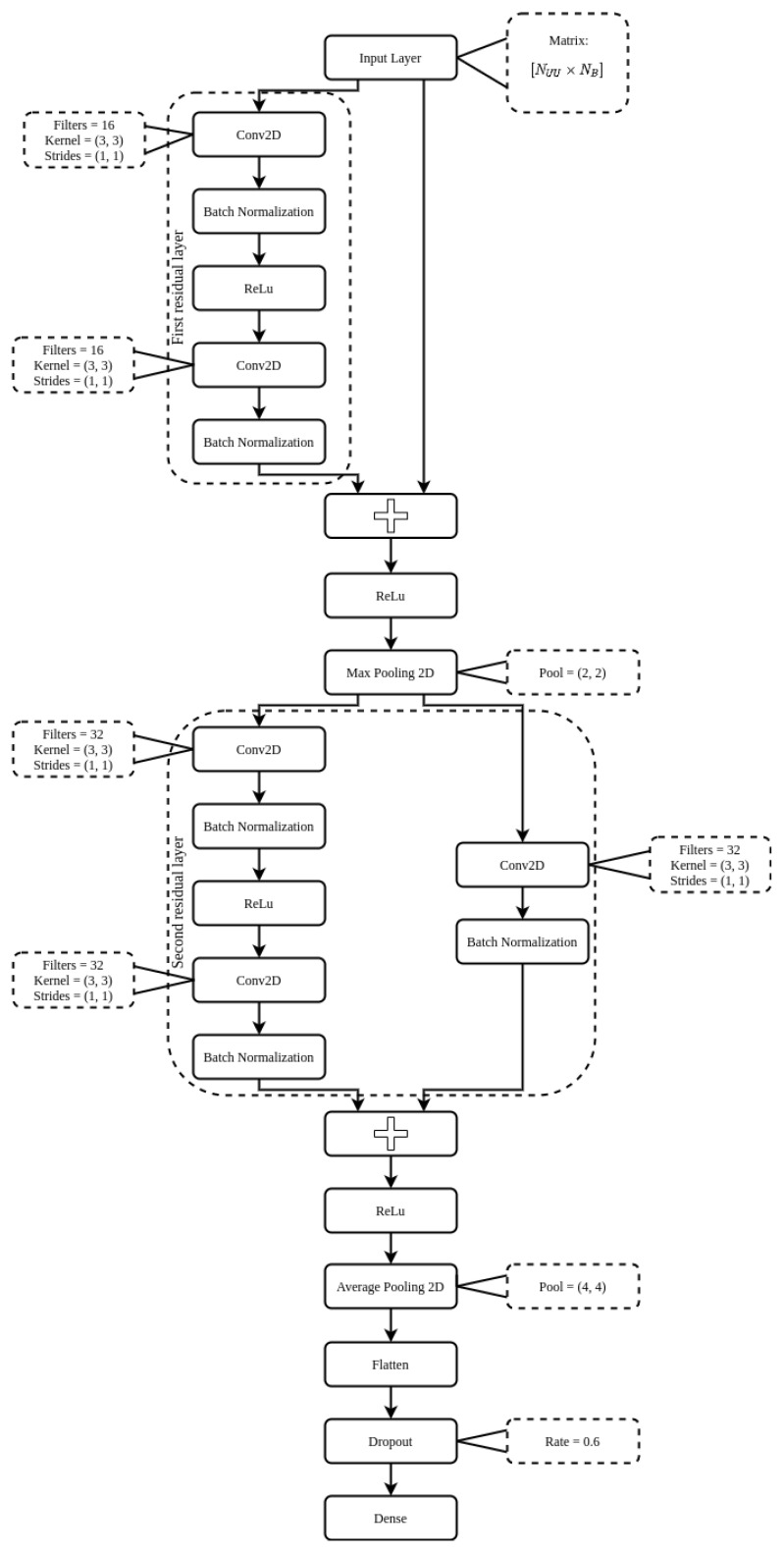
The proposed ResNet architecture.

The input data of ResNet are 2D matrices [$N_{UU}$×$N_{B}$], so the proposed architecture is as follows: (i) initially we have a residual layer composed of a convolutional layer 2D (*conv2D*), for feature extraction, followed by a *batch normalization* layer, which aims to make the network faster and more stable during the normalization process, and then the activation function *rectified linear unit* (ReLu). Then, we have another layer *conv2D* followed by a layer *batch normalization*, now, however, we add a *Add*, H(x)=F(x)+x, which has, in order to calculate the residual of the network, which really must be learned compared to what was already known from the input data, F(x)=H(x)−x, where F(x) is mapping of the learnable layers and *x* are the input data [34]. Finishing with the ReLu activation function; (ii) the next layer is a *max pooling 2D*, which aims to reduce the dimensionality of the layer’s input data and allow assumptions about the resources contained in the clustered sub-regions [34]; (iii) a second residual layer is applied, where we have a *conv2D* layer followed by a *batch normalization* and a ReLu, sequentially another *conv2D* and *batch normalization*. All this is in parallel with a *conv2D* and, also, a *batch normalization*. A *Add* calculates the residuals between the two *batch normalization* in parallel, then the activation function ReLu is applied [34]; (iv) the next layer is *average pooling 2D*, which is a dimensional stair reduction operation that calculates the average value for *patches* of a feature map [34]; (v) a *flatten* layer is used for data vectorization; (vi) followed by a *dropout* layer, which aims to avoid *overfitting* by randomly setting input units to 0 with a rate frequency at each step during training time; (vii) and finally a layer *dense*, which is a fully connected layer with activation function *softmax*, $y=\frac{e^{x}}{\sum e^{x}}$. Figure 2 presents the complete architecture of the proposed ResNet.

### 3.5. Metrics

**Figure 3:**
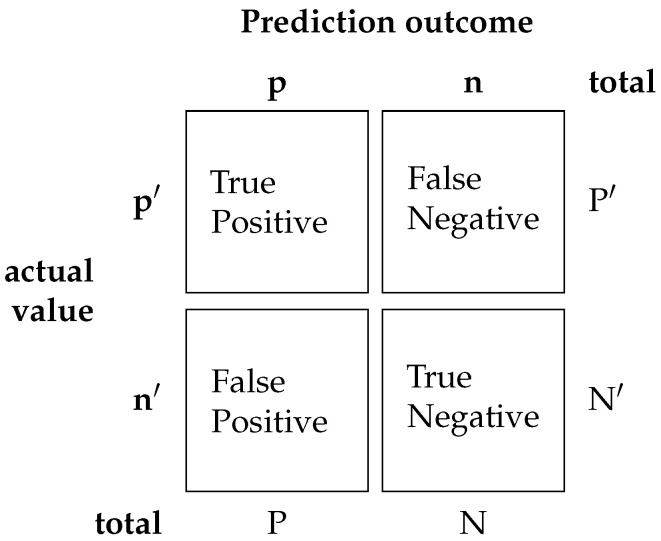
Exampleof confusion matrix.

To evaluate our models and our system in general we used some metrics, such:

Accuracy:
  (13) $$Accuracy=\frac{TP+TN}{TP+TN+FN+FP}$$
  where TP is true positive, TN is true negative, FP is false positive and FN is false negative.
Confusion matrix, Figure 3:

## 4. Experiments and Results

In this section, we will present the results and analyses on the proposed cooperative sensing approach using feature extraction, RF classifier for individual SS and the ResNet for wideband CSS. We compare the proposed method with usual machine learning and deep learning approaches in order to identify the contributions of the proposed method.

### 4.1. Dataset Generation

In the first step of the experiments, we generate the signals and extract features in order to highlight important proprieties to discriminate signals. In the generation of signals, we assume that several UU and a single LU move at a speed of v=3 km/h and their start position are randomly chosen in a given area of 250 m × 250 m, so that user’s location changes over a period time Δt=2 s. Moreover, the number of bands, $N_{B}$, is set to 16, where the bandwidth, $B_{W}$, is set to 10 MHz and $N_{B}$ is randomly chosen from 1 to 3, the LU can use up to 3 bands simultaneously. Furthermore, we assume that the P=23 dBm, $\beta =10^{3.453}$, α=3.8, σ=7.9 dB and the $N_{0}$ is randomly chosen between −114 and −174 dBm/Hz. The proportion of power leaked to adjacent bands, η, is set to 10 dBm, so the power leaked to adjacent bands is half of the power of the LU. For the experiment, we generate 150,000 instances divided 75% and 25% for training and testing, respectively, for each $N_{UU}$ that variate between one and twenty, totaling 750,000 instances for all experiments. We generate 1024 samples per second of the signal.

**Figure 4:**
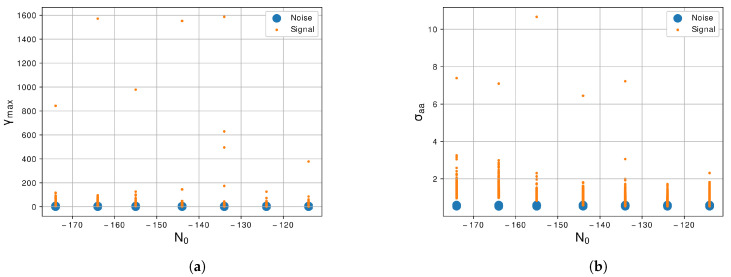
Graphic of the features (**a**) *γ_max_*, Equation (3), and (**b**) *σ_aa_*, Equation (4), with the variation of *N*_0_ of −114 dBm/Hz to −174 dBm/Hz.

After the generation of signals, in order to reduce the complexibility of the system, we propose extract features described by Equations (3) to (12). In Figure 4a, we can see the representation of the maximum value of the PSD of the normalized and centralized instantaneous amplitude, $\gamma_{max}$, of the two evaluated classes. We can notice that the noise signal and the LU signal are very correlated because in all levels of $N_{0}$ they presented a similar value of $\gamma_{max}$, which does not help the classifier in the correct identification of the classes. In Figure 4b, we show the representation of the standard deviation of the normalized and centralized instantaneous amplitude, $\sigma_{aa}$, of the two classes. In this feature, we can notice that the values of noise signal and LU signal present a low level of correlation, especially in lowest levels of $N_{0}$. We can say that the $\sigma_{aa}$ feature can differentiate the noise from the LU signal more easily.

**Figure 5:**
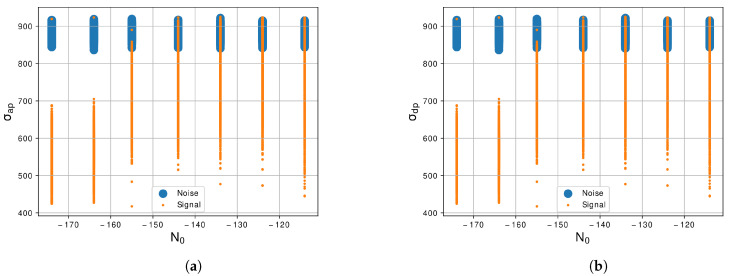
Graphic of the features (**a**) *σ_ap_*, Equation (5), and (**b**) *σ_dp_*, Equation (6), with the variation of *N*_0_ of −114 dBm/Hz to −174 dBm/Hz.

In Figure 5a, we can see the representation of the standard deviation of the centralized nonlinear absolute instantaneous phase, $\sigma_{ap}$, for the noise signal and the LU signal. In this case, we can notice that the difference between noise signal and LU signal is large, specially in the lowest level of $N_{0}$ where there is basically no intersection between the classes. Even in the highest level of $N_{0}$, there is still a large difference between noise signal and LU signal. So, this is also a feature that helps the classifier segregate the LU signal and the noise. The representation of standard deviation of the centralized direct nonlinear phase, $\sigma_{dp}$, Figure 5b, of the classes is very similar to the $\sigma_{ap}$ feature, in the highest level of $N_{0}$ there is just a small area of superposition of the classes, which facilitates the classifier to segregate the instances.

**Figure 6:**
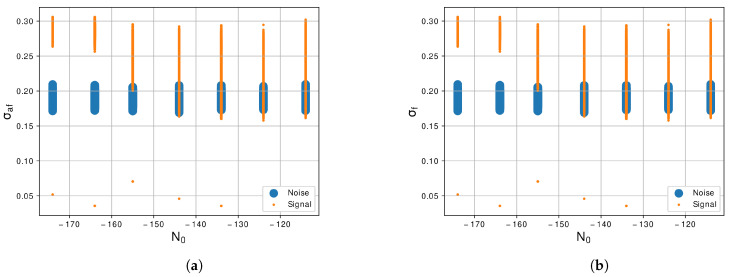
Graphic of the features (**a**) *σ_af_*, Equation (7), and (**b**) *σ_f_*, Equation (8), with the variation of *N*_0_ of −114 dBm/Hz to −174 dBm/Hz.

The standard deviation of normalized and centralized instantaneous frequency, $\sigma_{af}$, is shown in Figure 6a. We can notice that there is no superposition of values between the two classes in the lowest levels of $N_{0}$. Even in the highest levels, still there is a small area of intersection, which shows that the $\sigma_{af}$ is a promising resource to facilitate the process of classification. Very similar to the $\sigma_{af}$, the standard deviation of the absolute value of the normalized and centralized instantaneous frequency, σf, Figure 6b, shown a big differentiation between the classes, with a small area of correlation in the highest levels of $N_{0}$. This feature also demonstrate useful for distinguishes the noise from LU signal.

**Figure 7:**
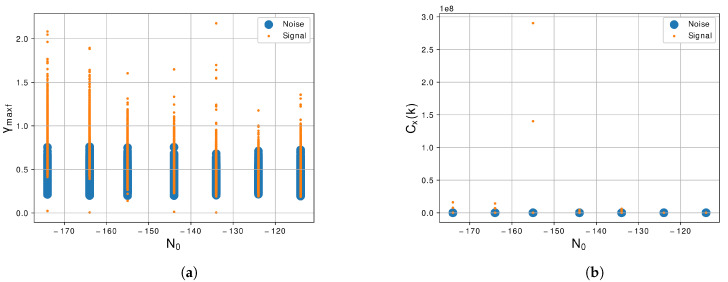
Graphic of the features (**a**) *γ_maxf_*, Equation (9), and (**b**) *C_x_(k)*, Equation (10), with the variation of *N*_0_ of −114 dBm/Hz to −174 dBm/Hz.

Figure 7a shows the scatter plot of the maximum PSD value of normalized and centralized instantaneous frequency, $\gamma_{maxf}$, for the noise signal and the LU signal. We can notice that this is a feature that has some level of correlation between the classes in all levels of $N_{0}$. Although, in the lowest levels of $N_{0}$, there is less superposition of the values of amplitude. In Figure 7b, we can see the maximum value of the discrete cosine transform, $max_{dct}$. We can notice that if the values of amplitude are very correlated, then the relevance of this feature for the classification algorithm is almost none.

**Figure 8:**
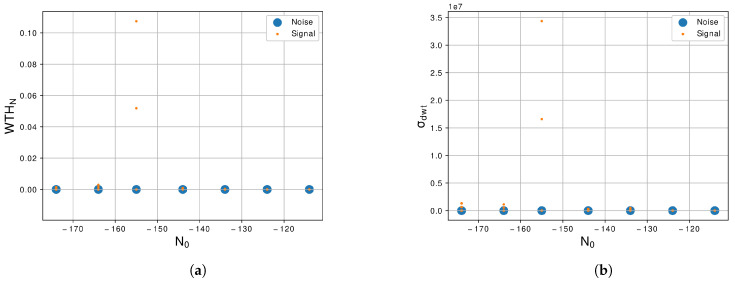
Graphic of the features (**a**) *WTH_N_*, Equation (11), and (**b**) *σ_dwt_*, Equation (12), with the variation of *N*_0_ of −114 dBm/Hz to −174 dBm/Hz.

The maximum value of the Walsh–Hadamard transform, $\sigma_{wht}$, is shown in Figure 8a. We notice that the behavior of this feature is very similar of the $max_{dct}$, the values of amplitude of this feature are very correlated, then this feature, also, does not represent much relevance for the classification algorithm. Furthermore, finally, the standard deviation of the discrete Wavelet transform, $\sigma_{dwt}$ is shown in Figure 8b. Furthermore, similar to the two previous features presented, $max_{dct}$ and $\sigma_{wht}$, the $\sigma_{dwt}$ presented a high level of correlation between the two classes due to the superposition of values of amplitude. So, $\sigma_{dwt}$ is also not demonstrated to be relevant to the classification task due the difficult to segregate classes.

After all this process of extract features, we can represent a signal evaluated in a vector with size ten, the number of features extracted. With this operation, we expect to reduce the complexity of the model required to classify the two classes, resulting in a reduction of time response, computational cost and energy. To discriminate this representation of signals in noise and LU signal, we propose a RF classifier and compare the results of the RF with some classical machine learning approaches. The output of the RF classifier is a binary answer, 1 if the LU is present in the channel evaluated, and 0 if it is not present. So for each UU, we will have a vector with size 16, that is the $N_{B}$ evaluated for UU, with the conditions of each one of these channels. All the methods presented, including the proposed RF classifier, were configured based on the default settings of the *scikit-learn* library.

**Figure 9:**
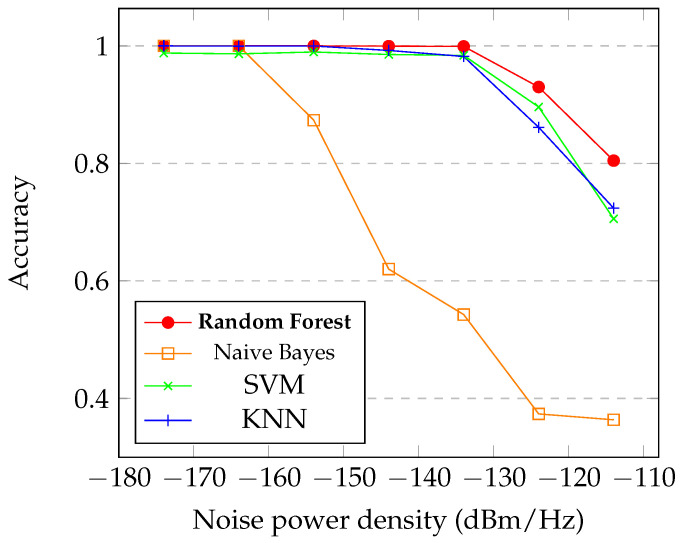
Graphic of the accuracy of the RF in comparison with classical machine learning approaches with $N_{0}$ between −114 dBm/Hz and −174 dBm/Hz.

In Figure 9, we can see the accuracy of the proposed RF classifier in comparison with the classical machine learning approaches. We can notice that the RF has the best results in the higher levels of $N_{0}$ and reaching 100% with lowest $N_{0}$. Comparing with the other methods, we can notice that kNN and SVM presented good results too, especially in the lowest levels of $N_{0}$. The Naive Bayes was the method with the lower accuracy response, achieving good results only in the lowest $N_{0}$ evaluated. The RF classifier obtained in the higher level of noise, −114 dBM/Hz, a accuracy superior to 80% and reaching more than 95% with $N_{0}$ inferior than −130 dBm/Hz.

**Figure 10:**
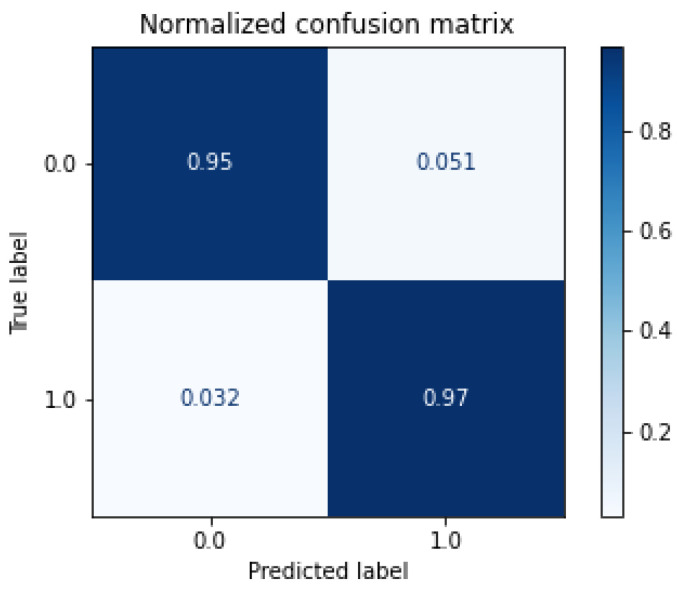
Confusion matrix of the RF Classifier with $N_{0}$ between −114 dBm/Hz and −174 dBm/Hz.

In Figure 10, we show the confusion matrix of the proposed RF classifier for the range of $N_{0}$ between −114 dBM/Hz and −174 dBM/Hz. We can notice that the RF model was capable of recognizing about 97% of true positives of LU signals and about 95% of noise, and the model misunderstood just a few instances, as we can see in the confusion matrix. These misunderstood instances were probably caused by signals with a high level of $N_{0}$.

### 4.2. Css Results

For the CSS approach, the proposed ResNet input matrix is composed of $N_{UU}$ and $N_{B}$ [$N_{UU}$×$N_{B}$], so we have a matrix 2D that contains the information of the conditions of 16 channels and the cooperation of $N_{UU}$ users. The proposed method is compared with other deep learning approaches such as CNN and RNN to show the benefits of ResNet in CSS taking into account the variation of $N_{0}$ with 10 UU cooperating. In these subsections, we also present the system response with the variation of $N_{UU}$ comparing with the CNN and RNN networks and taking into account the $N_{0}$ varying between −114 dBm/Hz and −174 dBM/Hz. Another analysis is made in relation to the processing time of the entire system with the proposed ResNet network and the other methods.

The parameters of the proposed ResNet is described as follow: in the *conv2D* residual layers we have a *kernel* [3,3] and *strides* [1,1], the parameter *padding* is set to *same*, the first and second residual layers extract 16 and 32 filters, respectively. After the first residual layer, there is a layer *max pooling 2D* with *pool size* of size [2,2], which reduces the output matrix size of the first residual layer by half. After the second residual layer there is a layer *average pooling 2D* with *pool size* of size [4,4], which reduces the size of the output matrix of the second residual layer by four times. Then, there is a *flatten* layer, which vectors the data, and a *dropout* layer with a rate of 0.6. Finally, there is a fully connected layer, *dense*, with activation function *softmax*, where the outputs are binary, representing whether or not there is presence of LU in the evaluated channels. The optimization function used is *Adam* with a learning rate equal to 0.001. The number of epochs is 100, and the *batch size* is 32.

The parameters of the CNN and RNN are described as follow: the projected CNN has two layers *conv2D*, with 8 and 16 filters, respectively. For these two layers, the ReLu activation function was applied. In sequence, a 2D *max pooling* is applied, followed by a *flatten* layer and a *dense* layer with 64 neurons with activation function *softmax*. The RNN has 3 fully connected layers with 100, 50 and 2 neurons, respectively. In the first two layers, the activation function is ReLu and the last layer has the *softmax* function as the activation function. The optimization function, learning rate, number of epochs and *batch size* are the same applied in the proposed ResNet.

**Figure 11:**
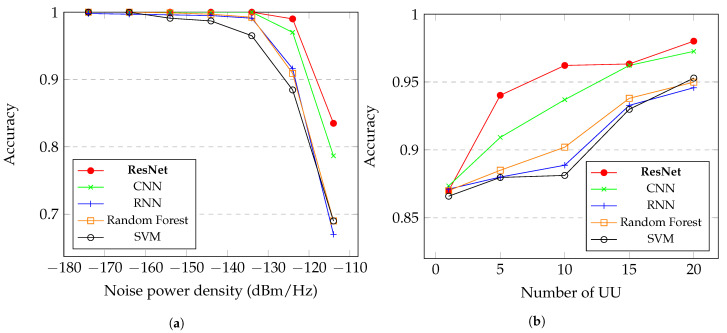
Graphics of the proposed ResNet in comparison with other machine learning and deep learning approaches with variation of (**a**) *N*_0_ and (**b**) *N_UU_*.

In Figure 11a, we present the accuracy of the proposed ResNet network in comparison with CNN and RNN networks. We can notice that the ResNet approach has the better response, especially in high levels of $N_{0}$ reaching more than 90% of accuracy when the $N_{0}$ is lower than −124 dBm/Hz. With the $N_{0}$ below −134 dBm/Hz, the accuracy is superior to 98%. These results take into consideration the cooperation of 10 UU in the system. In Figure 11b, we present the graphic of the response of the ResNet with the increase of the UU in the system. We can notice that when the $N_{UU}$ grows, the accuracy of the system also increases, and with 5 UU the accuracy of the proposed method reaches about 94%, while the others methods do not get to 92%. With 10 UU, the ResNet has a accuracy superior to 96%, and with 20 UU, the accuracy is about 98% with $N_{0}$ varying between −114 dBm/Hz and −174 dBm/Hz.

**Figure 12:**
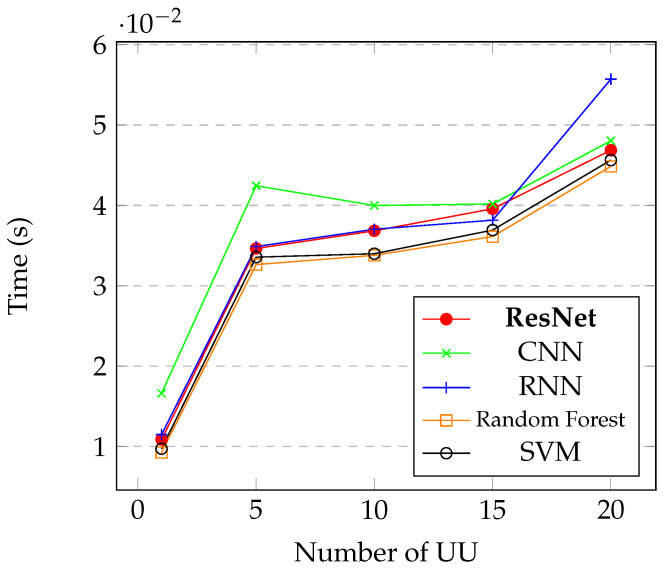
Time response of the entire system of the proposed ResNet model with other machine learning and deep learning approaches taking into consideration the variation of UU.

Another analysis that is important for CSS approach is the time response of the system. We can notice in Figure 12 that with the increase of UU, the time response grows, which is because the classification model has to be more robust to classify more UU in the system. We can see that even comparing with less complex architectures of neural networks, the ResNet can yet give, in most cases, the lower time response.

## 5. Discussion

In this paper, we approach a CSS based on a ResNet using feature extractor and RF classifier. In the first step of the proposal methodology, we generate signals based on the Equations (1) and (2). After we extract features, similar to proposal in [30], but instead 29 as in [30] we propose 10 as describe by the Equations (3) to (12), which are spectral and transform features selected to represent the signals due the capacity of highlight important characteristics of the signals. This feature extraction has the purpose of reducing complexity and computational cost. With this new representation of the signal, a RF classifier is proposed to identify if this representation is a LU signal or a noise signal. Then, a matrix with the information of channels of many UU is used as input to a model that can identify in wideband the presence of LU in many channels.

The generation of the signals was done in a similar way as proposed by [28] with some differences. The first and most relevant is the range of $N_{0}$ applied in your studies, they vary between −154 dBm/Hz and −174 dBm/Hz, while we apply a range of −114 dBm/Hz and −174 dBm/Hz. Our proposal contemplates signal conditions with greater noise influence, which gave us a better perspective of how our methodology responds in relation to more stressful signal propagation environments. Another change in signal generation compared to [28] is the UU and LU locomotion area, in [28] your users can move in an area of 200 m × 200 m while we configure this area at 250 m × 250 m. Signal degradation is related to the distance between UU and LU, so in a larger locomotion area, the signal is expected to be more influenced by noise. Our signals generation also takes into consideration the low quantity of UU in the system, in our experiment we simulate the cooperation of 20 UU in the max, which due the founded results, gives to our methodology even more reliability.

Regarding the feature extractor, we extract from the generated signals 10 features of the 29 proposed by [30]. The first feature is the $\gamma_{max}$, Equation (3), we notice in this feature that the noise and the LU signal are correlated as we can see in the graphic of Figure 4a, so this feature is not a prior for segregate the two classes. The second feature is the $\sigma_{aa}$, Equation (4), and this feature shows a better response than the previous and can differentiate noise and LU signal more easily as shown in Figure 4b. The next feature is the $\sigma_{ap}$, Equation (5), and the differences between the two classes are large, facilitating the segregation of the classes as shown in Figure 5a. The forty feature, shown in Figure 5b, is the $\sigma_{dp}$, Equation (6), and similar to the previous feature there is a large difference between the two classes. The fifth and sixth features are the $\sigma_{af}$ and σf, Equations (7) and (8), shown in Figure 6a,b also present a good difference between the two classes. The next feature is the $\gamma_{maxf}$, Equation (9), and shows some level of correlation in all levels of $N_{0}$ as we can see in Figure 7a, even in lowest level of $N_{0}$, which still have some superposition between the classes. Furthermore, the last three features, Figure 7b and Figure 8a,b, show a high level of correlation and may not be relevant in the classification task. In this study, we conclude that the best features to represent the signals are the $\sigma_{f}$, $\sigma_{af}$, $\sigma_{dp}$, $\sigma_{ap}$ and $\sigma_{aa}$, due to the lowest level of superposition between the two classes in all levels of $N_{0}$. In [30], they obtained good results with the approach with 29 features, for our methodology 10 from this 29 were enough to obtained excellent results, a study to optimize even more of these features could be a future work.

The RF classifier is proposed to evaluate the individual conditions of each channel. We compare the RF approach with three classical machine learning algorithms and the proposed RF obtained the better result especially in highest levels of $N_{0}$. With the $N_{0}$ in −114 dBm/Hz the RF obtained more than 80% of accuracy, already with −134 dBm/Hz the accuracy achieve about 98% of accuracy, in the lowest $N_{0}$, the accuracy is 100% in the correct classification of the noise and LU signal. In the graphic of Figure 9, we can notice the superior performance of the RF classifier in the highest level of noise if compared with the others classical approaches. In comparison, for instance, the Naive Bayes obtained poor performance in the high level of $N_{0}$, the other approaches, SVM and KNN, obtained good performance, but do not achieve the level of performance of the RF classifier. In the confusion matrix of the proposed RF, Figure 10, we can notice that the RF obtained a excellent result. In [31], the authors obtained 91% of accuracy in the identification of spectrum holes based in energy detection, in our proposed method we obtained about 96% of accuracy.

The proposed method for CSS based on ResNet has a better response than the other compared methods. In Figure 11a, we can see that the proposed ResNet performed better especially at higher $N_{0}$, with −114 dBm/Hz, for example, the ResNet precision was about 83% while CNN and RNN obtained accuracy of less than 80%, this with the cooperation of 10 UU. The proposed method achieves more than 98% accuracy with $N_{0}$ below −130 dBm/Hz. In Figure 11b, we show the increase of accuracy of the proposed method with the grow of the $N_{UU}$, we can notice that the ResNet obtained a better performance that the others methods, reaching about 98% with 20 UU in the system. In [28], they used a CNN together with energy detector to CSS, they results was taking in consideration a lower level of $N_{0}$ and with much larger $N_{UU}$ in the system, we obtained high accuracy with a higher level of $N_{0}$ and less $N_{UU}$ in the system. Furthermore, in [35], for example, they achieve about 95% of accuracy with the cooperation of 20 UU, while we obtained about 98%. The time response of the system also has been taken in consideration, in Figure 12 we shown the computation time in seconds, we can see that the proposed method was faster in all most points, and maintained the time lower than 0.05 seconds even with 20 UU in the system. In [28], they obtained a faster response with 20 UU than our proposed method, while in [35] they obtained a bigger response time for testing in some approaches. For future works, we propose an increase of the $N_{UU}$ to evaluate the proposed system.

Comparing the proposed method with classical approaches, such as RF classifier and the SVM, we can notice that the ResNet achieved better accuracy in all levels of $N_{0}$ with the presence of 10 UU in the system, as we can see in Figure 11a. We can notice that the classical methods had performance similar to the RNN classifier. Making a comparison related to the increase of UU in the system, we can say that the proposed ResNet also achieve better accuracy than the classical machine learning approaches. We can notice that the RF classifier obtained better performance that the RNN, and the SVM obtained similar performance that the RNN, but with 20 UU the SVM obtained better performance then both RF and RNN, as showed in Figure 11b. In Figure 12, we compared the time response of the system with different models, we can notice that the classical methods obtained better response time that the proposed method, but are not worth the use of the classical methods instead of the proposed ResNet, due to the high difference of accuracy and the little difference of time response.

## 6. Conclusions

The present study presents a framework for CSS based on ResNet using feature extractor and RF classifier. The feature extractor was able to reduce the complexity of the signals and enable an RF classifier to be sufficient to classify signals with high precision and with high noise influence, achieving about 98% accuracy with $N_{0}$ in −134 dBm/Hz. Extensive simulations and numerical results have shown that the proposed ResNet for CSS can achieve greater accuracy even in environments where signals are heavily influenced by noise, around 98% with $N_{0}$ at −130 dBm/Hz and with 10 UU in the system. Another point is related to system-wide response time, we showed that we can get a faster response even with considerable $N_{UU}$, with response times less than 0.05 s.

The spectral and transformation features extracted really showed to be effective in highlight singular characteristics from the signals received by the UU. This could be verified by the performance of the proposed RF classifier, which achieves a high success rate even at high levels of $N_{0}$, which shows that the combination of these two methods is efficient to recognize LU in the evaluated channels even under adverse conditions. Another contribution of this paper is related to the deep neural network proposed to CSS. For instance, comparing our results with the proposed by [28] we can notice that our proposed ResNet achieve similar accuracy rate in higher levels of $N_{0}$ and without the need of so many UU in the system. Then, all our pipeline showed a good level of reliability in adverse channel conditions, which is needed in high data transmission rate systems, such the new generations of communication systems.

As future work, improvements can be made regarding resource optimization. A study of the performance of the proposed method by reducing the number of extracted features must be done, since we realized that some of the features we proposed to extract from the signals were demonstrated to have a high level of correlation between the two classes. This can increase performance and reduce system response time. Another future work is related to $N_{UU}$, in the literature they propose a large number of UU in the system, but in our proposed method only 20 were tested in the experiments. It would be ideal to also rate the system with a higher $N_{UU}$.

## Data Availability

In this article, we generate a database as described in Section 3.3. The raw data is around 25 GB. Data after feature extraction is approximately 150 MB. All data can be shared. If you are interested, please contact the corresponding authors M.V. and W.S..

## References

[1] Akyildiz I.F., Lee W.-Y., Vuran M.C., Mohanty S. (2008). A survey on spectrum management in cognitive radio networks. IEEE Commun. Mag..

[2] Akyildiz I.F., Lo B.F., Balakrishnan R. (2011). Cooperative spectrum sensing in cognitive radio networks: A survey. Phys. Commun..

[3] Subhedar M., Birajdar G. (2011). Spectrum Sensing Techniques in Cognitive Radio Networks: A Survey. Int. J. Next-Gener. Netw..

[4] Singh J., Shukla A. (2020). Spectrum Sensing in MIMO Cognitive Radio Networks Using Likelihood Ratio Tests with Unknown CSI. Intelligent Communication, Control and Devices.

[5] Shellhammer S.J. (2008). Spectrum sensing in IEEE 802.22. Iapr Wksp. Cogn. Info. Process..

[6] Zeng Y., Liang Y.-C., Hoang A.T., Zhang R. (2010). A Review on Spectrum Sensing for Cognitive Radio: Challenges and Solutions. EURASIP J. Adv. Signal Process..

[7] Jin M., Guo Q., Xi J., Li Y., Yu Y., Huang D. (2015). Spectrum Sensing Using Weighted Covariance Matrix in Rayleigh Fading Channels. IEEE Trans. Veh. Technol..

[8] Arjoune Y., Kaabouch N. (2019). A Comprehensive Survey on Spectrum Sensing in Cognitive Radio Networks: Recent Advances, New Challenges, and Future Research Directions. Sensors.

[9] Gupta M.S., Kumar K. (2019). Progression on spectrum sensing for cognitive radio networks: A survey, classification, challenges and future research issues. J. Netw. Comput. Appl..

[10] Jain P.P., Pawar P.R., Patil P., Pradhan D. (2019). Narrowband Spectrum Sensing in Cognitive Radio Detection Methodologies. Int. J. Comput. Sci. Eng..

[11] Arjoune Y., El Mrabet Z., El Ghazi H., Tamtaoui A. Spectrum sensing: Enhanced energy detection technique based on noise measurement. Proceedings of the 2018 IEEE 8th Annual Computing and Communication Workshop and Conference (CCWC).

[12] Liu X., Sun C., Zhou M., Wu C., Peng B., Li P. (2020). Reinforcement learning-based multislot double-threshold spectrum sensing with Bayesian fusion for industrial big spectrum data. IEEE Trans. Ind. Inform..

[13] Ilyas I., Paul S., Rahman A., Kundu R.K. Comparative evaluation of cyclostationary detection based cognitive spectrum sensing. Proceedings of the 2016 IEEE 7th Annual Ubiquitous Computing, Electronics & Mobile Communication Conference (UEMCON).

[14] Kabeel A.A., Hussein A.H., Khalaf A.A., Hamed H.F. (2019). A utilization of multiple antenna elements for matched filter based spectrum sensing performance enhancement in cognitive radio system. AEU Int. J. Electron. Commun..

[15] Dannana S., Chapa B.P., Rao G.S. (2018). Spectrum Sensing Using Matched Filter Detection. Intelligent Engineering Informatics.

[16] Chen A.-Z., Shi Z.-P. (2018). A Real-Valued Weighted Covariance-Based Detection Method for Cognitive Radio Networks With Correlated Multiple Antennas. IEEE Commun. Lett..

[17] Sun H., Nallanathan A., Wang C.-X., Chen Y. (2013). Wideband spectrum sensing for cognitive radio networks: A survey. IEEE Wirel. Commun..

[18] Quan Z., Cui S., Sayed A.H., Poor H.V. (2008). Optimal Multiband Joint Detection for Spectrum Sensing in Cognitive Radio Networks. IEEE Trans. Signal Process..

[19] Sharma K., Sharma A. Design of Cosine Modulated Filter Banks exploiting spline function for spectrum sensing in Cognitive Radio applications. Proceedings of the 2016 IEEE 1st International Conference on Power Electronics, Intelligent Control and Energy Systems (ICPEICES).

[20] Hoyos E.A., Parra O.J.S., Muñoz W.Y.C., Sanabria L.F.M. (2020). Centralized sub-Nyquist wideband spectrum sensing for cognitive radio networks over fading channels. Comput. Commun..

[21] Vasavada Y., Prakash C. (2020). Sub-Nyquist Spectrum Sensing of Sparse Wideband Signals Using Low-Density Measurement Matrices. IEEE Trans. Signal Process..

[22] Solanki S., Dehalwar V., Choudhary J. (2021). Deep Learning for Spectrum Sensing in Cognitive Radio. Symmetry.

[23] Varun M., Annadurai C. (2021). PALM-CSS: A high accuracy and intelligent machine learning based cooperative spectrum sensing methodology in cognitive health care networks. J. Ambient. Intell. Humaniz. Comput..

[24] Shachi P., Sudhindra K.R., Suma M.N. Convolutional neural network for cooperative spectrum sensing with spatio-temporal dataset. Proceedings of the 2020 International Conference on Artificial Intelligence and Signal Processing (AISP).

[25] Ghasemi A., Sousa E.S. (2007). Spectrum sensing in cognitive radio networks: The cooperation-processing tradeoff. Wirel. Commun. Mob. Comput..

[26] Zhang W., Mallik R.K., Letaief K.B. Cooperative spectrum sensing optimization in cognitive radio networks. Proceedings of the 2008 IEEE International Conference on Communications.

[27] Nasser A., Chaitou M., Mansour A., Yao K.C., Charara H. (2021). A Deep Neural Network Model for Hybrid Spectrum Sensing in Cognitive Radio. Wirel. Pers. Commun..

[28] Lee W., Kim M., Cho D.-H. (2019). Deep Cooperative Sensing: Cooperative Spectrum Sensing Based on Convolutional Neural Networks. IEEE Trans. Veh. Technol..

[29] Shawel B.S., Woledegebre D.H., Pollin S. Deep-learning based cooperative spectrum prediction for cognitive networks. Proceedings of the 2018 International Conference on Information and Communication Technology Convergence (ICTC).

[30] Furtado R.S., Torres Y.P., Silva M.O., Colares G.S., Pereira A.M.C., Amoedo D.A., Valadao M.D.M., Carvalho C.B., da Costa A.L.A., Junior W.S.S. Automatic Modulation Classification in Real Tx/Rx Environment using Machine Learning and SDR. Proceedings of the 2021 IEEE International Conference on Consumer Electronics (ICCE).

[31] Mishra A., Dehalwar V., Jobanputra J.H., Kolhe M.L. Spectrum hole detection for cognitive radio through energy detection using random forest. Proceedings of the 2020 International Conference for Emerging Technology (INCET).

[32] Hazza A., Shoaib M., Alshebeili S.A., Fahad A. An overview of feature-based methods for digital modulation classification. Proceedings of the 2013 1st International Conference on Communications, Signal Processing, and their Applications (ICCSPA).

[33] Couronné R., Probst P., Boulesteix A.-L. (2018). Random forest versus logistic regression: A large-scale benchmark experiment. BMC Bioinform..

[34] Kim C., Park D., Lee H.-N. (2020). Compressive Sensing Spectroscopy Using a Residual Convolutional Neural Network. Sensors.

[35] Shi Z., Gao W., Zhang S., Liu J., Kato N. (2020). Machine Learning-Enabled Cooperative Spectrum Sensing for Non-Orthogonal Multiple Access. IEEE Trans. Wirel. Commun..

